# Proteomic Analysis of Cellular Response Induced by Multi-Walled Carbon Nanotubes Exposure in A549 Cells

**DOI:** 10.1371/journal.pone.0084974

**Published:** 2014-01-14

**Authors:** Li Ju, Guanglin Zhang, Xing Zhang, Zhenyu Jia, Xiangjing Gao, Ying Jiang, Chunlan Yan, Penelope J. Duerksen-Hughes, Fanqing Frank Chen, Hongjuan Li, Xinqiang Zhu, Jun Yang

**Affiliations:** 1 Collaborative Innovation Center for Diagnosis and Treatment of Infectious Diseases, State Key Laboratory for Infectious Diseases Diagnosis and Therapy, The First Affiliated Hospital, Zhejiang University School of Medicine, Hangzhou, China; 2 Department of Toxicology, Zhejiang University School of Public Health, Hangzhou, China; 3 Zhejiang Academy of Medical Sciences, Hangzhou, China; 4 Department of Basic Science, Division of Biochemistry, Loma Linda University School of Medicine, Loma Linda, California, United States of America; 5 Life Science Division, Lawrence Berkeley National Laboratory, Berkeley, California, United States of America; 6 Hangzhou Normal University School of Public Health, Hangzhou, China; 7 College of Biotechnology, Zhejiang Agriculture and Forestry University, Hangzhou, China; National Institutes of Health, United States of America

## Abstract

The wide application of multi-walled carbon nanotubes (MWCNT) has raised serious concerns about their safety on human health and the environment. However, the potential harmful effects of MWCNT remain unclear and contradictory. To clarify the potentially toxic effects of MWCNT and to elucidate the associated underlying mechanisms, the effects of MWCNT on human lung adenocarcinoma A549 cells were examined at both the cellular and the protein level. Cytotoxicity and genotoxicity were examined, followed by a proteomic analysis (2-DE coupled with LC-MS/MS) of the cellular response to MWCNT. Our results demonstrate that MWCNT induces cytotoxicity in A549 cells only at relatively high concentrations and longer exposure time. Within a relatively low dosage range (30 µg/ml) and short time period (24 h), MWCNT treatment does not induce significant cytotoxicity, cell cycle changes, apoptosis, or DNA damage. However, at these low doses and times, MWCNT treatment causes significant changes in protein expression. A total of 106 proteins show altered expression at various time points and dosages, and of these, 52 proteins were further identified by MS. Identified proteins are involved in several cellular processes including proliferation, stress, and cellular skeleton organization. In particular, MWCNT treatment causes increases in actin expression. This increase has the potential to contribute to increased migration capacity and may be mediated by reactive oxygen species (ROS).

## Introduction

Nanomaterials, with sizes ranging from 1 to 100 nm in one or more dimensions, are the core of an emerging technological revolution [1], [2]. Multi-walled carbon nanotubes (MWCNT), discovered by Iijima in 1991, is one of the most common nanomaterials in use [3]. Structurally, MWCNT consists of several concentric graphene sheets which can be produced in laboratories, and can even be found in the particulate matter from ordinary combustion of fuel gases [4], [5]. Due to its outstanding physicochemical and mechanical properties such as high tensile strength, ultra-light weight, thermal and chemical stability, as well as excellent semi-conductive electronic properties, MWCNT has been a highly desirable material in various sectors including electronics, aerospace, chemicals, construction and pharmaceuticals [6]. MWCNT has also being developed for a range of biomedical applications such as miniaturized biosensors, or for targeted drug delivery and tissue engineering [7], [8]. However, the wide application of MWCNT has raised serious concerns about their possible impact on safety for human health and the environment. Human may be exposed to MWCNT through inhalation, ingestion, or skin uptake, and when MWCNT interacts with biological systems, adverse biological effects might be generated.

Many studies have been conducted over the past several years to evaluate the toxicological effects of MWCNT. However, existing data are frequently contradictory. For example, MWCNT was able to induce the time- and dose-dependent cytotoxicity in several cell lines, leading to the release of proinflammatory cytokines, cell cycle arrest and apoptosis [9]–[14]. Due to its fibrous- or microtube-like structure, genotoxic damage such as chromosomal aberrations, DNA strand breakages and micronuclei were also found in cells after MWCNT treatment [9], [15]–[18]. Several studies have suggested that reactive oxygen species (ROS) may be responsible for the cytotoxicity and genotoxicity of MWCNT[19]–[21], and signaling pathways such as NF-κB, AP-1, p38/ERK-MAPK cascades have also been implicated [14], [22]. However, some other reports reveal no cytotoxicity following MWCNT treatment [20], [23]. For instance, MWCNT demonstrates no sign of acute toxicity on cell viability and could not induce any inflammatory mediators, such as NO, TNF-alpha and IL-8 in either rat macrophages (NR8383) or in human lung adenocarcinoma A549 cells [23]. Although the exact reason for the different biological effects of MWCNT is still unknown, it is believed that the size and shape of the nanomaterials, the presence of trace amounts of metals, and the cell types examined may all contribute to the observed differences [23]–[25].

Similar contradictory results were also reported in *in vivo* studies. For example, it has been shown that MWCNT induces pulmonary inflammation, granuloma, fibrosis, and mesothelioma in experimental models [26]–[30]. MWCNT could also alter the systemic immune function in mice [31]–[35], and individuals with pre-existing allergic inflammation may be susceptible to airway fibrosis from inhaled MWCNT [36]. Furthermore, recent observations suggest that the nervous system is vulnerable to MWCNT as well [37]–[39]. On the other hand, there are also reports showing no inflammation or cancer occurrence after MWCNT exposure in rats [40], [41].

As noted above, the potential large-scale exposure of humans to the biological effects of MWCNT requires a much better understanding of the risks and mechanisms involved, as well as a clarification of the origin of these contradictory results. To this end, the cytotoxic and genotoxic effects of MWCNT on A549 cells were examined, followed by application of a proteomics-based approach. By screening and identifying the differentially expressed proteins, we further investigated the possible roles of such proteins in MWCNT-induced toxicity. As reported here, MWCNT induces cytotoxicity in A549 cells only at relatively high concentrations and longer exposure time. Within a relatively low dosage range (30 µg/ml) and short time period (24 h), MWCNT treatment of A549 cells does not induce significant cytotoxicity, cell cycle arrest, apoptosis, or DNA damage. However, at these low doses and times, proteomic analysis reveals that MWCNT causes significant protein expression changes, and that differentially expressed proteins are involved in cellular processes such as proliferation, metabolism, and organization of the cellular skeleton.

## Materials and Methods

### MWCNT preparation

MWCNT was provided by Dr. F. Chen (Lawrence Berkeley National Laboratory, Berkeley, CA), and it was synthesized with a chemical vapor deposition (CVD) method [14]. The detailed physicochemical characterizations of MWCNT were described in our previous study [12]. The sterile raw material was suspended in 1640 culture medium (Gibco, Grand Island, NY, USA) containing 10% fetal calf serum and then the suspension was sonicated at 180 W for 30 cycles, with 10 s ultrasonication and 5 s pause using an ultrasonic disrupter (JY92-IIN, Scientz, Ningbo, China). The suspensions were always prepared fresh prior to use.

### Cell culture and Cytotoxicity analysis

Human lung adenocarcinoma A549 cells, obtained from the ATCC (CCL-185), were routinely subcultured in 1640 culture medium containing 10% newborn calf serum, 100 U/ml penicillin, 125 µg/ml streptomycin and 0.03% glutamine at 37°C in 5% humidified CO_2_. MWCNT was added to cell media at different concentrations (0, 0.3, 3, 30 and 300 µg/ml) for various times (0, 2, 12 and 24 h). The effects of MWCNT on cell viability were examined by trypan blue exclusion as described previously [42]. Briefly, A549 cells were cultured in 24-well plates and subjected to various treatments. Then the cells were harvested and mixed with an equal volume of 0.4% (w/v) trypan blue solution prepared in PBS. The number of trypan blue-excluding cells was determined using a hemocytometer, and cell viability was calculated as the ratio of viable cells in treatment group/viable cells in control group. Experiments were performed in triplicate.

### Flow cytometry analysis of apoptosis and the cell cycle

A549 cells were collected after MWCNT treatment, and washed twice with PBS. For apoptosis detection, the cells were suspended in 400 µl binding buffer and incubated with 5 µl Annexin V-FITC (MultiSciences, Hangzhou, China) and 5 µl PI in the dark at 37°C for 15 min. Cells were put on ice until analysis. The ratio of apoptotic cells was measured using a Beckman Coulter Epics XL-MCL device (Fullerton, CA, USA).

For cell cycle detection, cells were fixed in 70% ethanol for at least 2 h. After fixation, cells were centrifuged at 200 g for 5 min, and the cell pellet suspended in 500 µl propidium iodide (PI)/Triton X-100 staining solution containing RNase A for 15 min at 37°C. Cell cycle was assessed using a Beckman Coulter Epics XL-MCL device.

### Immunofluorescence microscopy to detect genotoxicity of MWCNT

Immunofluorescence microscopy to observe the formation of γH2AX foci and distribution of actin was conducted essentially as described previously with slight modification [42]. Briefly, 1-2×10^5^ cells were seeded into glass-bottom 6-well plates. After treatment, cells were fixed in 4% paraformaldehyde for 15 min, washed with PBS once, and permeabilized in 0.2% Triton X-100 for 5 min. After blocking for 1 h, samples were incubated with a mouse monoclonal anti-γH2AX antibody (1∶3000) (Upstate Technology, Lake Placid, NY) overnight at 4°C, followed by incubation with FITC-conjugated goat-anti-mouse secondary antibody (1∶500) (Beijing Zhongshan Biotechnology Co., China) for 1 h. To stain the nuclei, Hoechst33258 (Sigma, St. Louis, USA) was added to the cells and incubated for another 15 min. For a positive control, genotoxin cis-diammine-dichloro-platinum (Pt) (Sigma, St. Louis, USA) was chosen for its ability to induce γH2AX foci formation [43]. For actin observation, cells were incubated with Phalloidin (Invitrogen, Carlsbad, USA) for 30 min and Hoechst33258 for 15 min in the dark. The glass-bottom 6-well plates were observed using a LSM710 Laser Scanning Confocal Microscope (Zeiss, Jena, Germany).

### Comet assay

The neutral comet assay was conducted as described previously [42]. First, the fully frosted microscope slides were covered with 100 µl of 0.65% normal melting point agarose and immediately covered with a coverslip. Slides were placed on ice for 8 min to allow the agarose to solidify. Next, the coverslips were removed, and the first agarose layer was covered with the cell suspension (1×10^6^ cells in 15 µl PBS were mixed with 75 µl of 0.65% low melting point agarose). After replacing the coverslips, the slide was allowed to solidify on ice for 8 min. Another layer of agarose (75 µl of 0.65% low melting point agarose) was then added as described above. Finally, the coverslips were removed, and the slides were immersed in the lysis buffer (2 M NaCl, 30 mM EDTA, 10 mM Tris, with 1% Triton X-100 and 10% DMSO added just before use, pH 8.2–8.5) for 2 h at 4°C. The slides were removed from the lysis buffer, washed for 10 min in 0.5×TBE, and transferred to an electrophoresis chamber. After equilibration in 0.5×TBE for 20 min, electrophoresis was conducted at 25 V for 20 min. The slides were then washed in a neutralization buffer (0.4 M Tris, pH 7.5) 3 times for 5 min. The slides were drained and stained with gel-red, and observed with a fluorescent microscope. Single cell images were captured and analyzed using an Olympus AX70 (Olympus, Japan) immunofluorescent microscope, and tail moment was scored using CometScore software (TriTek Corporation, Southern California, USA).

### 2-Dimentional electrophoresis (2-DE)

2-DE was conducted as previously described [44]. Briefly, 200 µg total cellular proteins were loaded onto 24 cm, pH 3–10 linear immobilized pH gradient strips (Bio-Rad, CA, USA) for isoelectric focusing (IEF). After 12 h of rehydration, the strips were transferred to the IEF cell. The parameters were set as follows: 250 V for 30 min, step; 500 V for 1 h, step; 1000 V for 3 h, gradient; 10,000 V for 6 h, gradient; 10,000 V until 80,000 Vhr, step. After IEF was completed, the strips were equilibrated and the second dimension was performed by vertical 12% SDS-PAGE. Gels were stained using silver staining and scanned with a Bio-Rad GS-800 scanner. Images were analyzed by PDQuest software Version 7.4.0 (Bio-Rad, Hercules, USA). A statistical analysis was performed using the Student's t-test. Proteins with significant differences (1.5 fold change, *P*<0.05) were selected for MS identification.

### LC-MS/MS identification

Differentially expressed protein spots were manually cut from the silver-stained gels and were destained with a solution of 30 mM potassium ferricyanide and 100 mM sodium thiosulfate, followed by dehydration in acetonitrile, and dried in vaccum centrifuge for 30 min. The gels were subsequently rehydrated in 10–20 µl of a proteomics-grade trypsin solution (20 µl/ml in 40 mM NH_4_HCO_3_ plus 9% acrylonitrile) and incubated at 37°C for 6 to 8 h. Peptides were extracted twice by adding 30 µl of a solution containing 50% acrylonitrile and 5% trifluoroacetic acid (TFA). The extracted solutions were lyophilized in a vacuum centrifuge. All digested peptide mixtures were separated by reverse phase (RP) HPLC followed by tandem MS analysis. RP-HPLC was performed using a Surveyor LC system (Thermo Finnegan, San Jose, CA) on a C18 reverse phase column (RP, 180 µm×150 mm, BioBasic C18, 5 µm, Thermo Hypersil-Keystone). Mobile phase A (0.1% formic acid in HPLC-grade water) and mobile phase B (0.1% formic acid in acetonitrile) were selected. Peptide mixture was loaded onto the column and separation of the peptides was performed at a flow rate of 1.5 µl/min using a gradient of 2–80% solution B for 60 min. The effluent from the reverse phase column was analyzed by an ESI mass spectrometer (LCQ Deca XP; Thermo Finnigan, San Jose, CA). The MS analysis was preformed with one full MS scan followed by 10 MS/MS scans on the 10 most intense ions from the MS spectrum with the dynamic exclusion settings: repeat count 2, repeat duration 30 s, exclusion duration 90 s. The acquired MS/MS spectra were automatically searched against database for human proteins (National Center for Biotechnology Information's human-subset, nonredundant protein database, 07-27-10) using the Bioworks Browser rev. 3.1 (Thermo Electron, San Jose, CA). Protein identification results were extracted from SEQUEST out files with BuildSummary. The peptides were constrained to be tryptic and up to two missed cleavages were allowed. Carbamidomethylation of cysteines were treated as a fixed modification, whereas oxidation of methionine residues was considered as variable modifications. The mass tolerance allowed for the precursor ions was 2.0 Da and fragment ions was 0.8 Da, respectively. The protein identification criteria were based on Delta CN (≥0.1) and cross-correlation scores (Xcorr, one charge≥1.9, two charges ≥2.2, three charges ≥3.75).

### Western blot analysis

Equal amount of proteins were loaded and separated by 10% SDS-PAGE, and then transferred to PVDF membranes in transfer buffer (25 mM Tris, 200 mM Glycine, 20% Methanol v/v). The membranes were blocked with 5% BSA in TBST (Tris 20 mM, NaCl 137 mM, Tween-20 0.1%, pH 7.6) for 1 h at room temperature. After washing with TBST, the membranes were incubated in primary antibody at 4°C overnight followed by incubation with the secondary antibody for 1 h at room temperature. Antibodies used included Hsp27 (Bioworld, diluted 1∶1000), actin (Santa Cruz, diluted 1∶5000), 14-3-3ε (Bioworld, diluted 1∶1000), and Secondary Antibodies (Multisciences, diluted 1∶5000). The protein bands were scanned using a FluorChem FC2 Imaging System (Alpha, San Antonio, USA). GAPDH (Santa Cruz, diluted 1∶3000) was employed as an internal control.

### Measurement of intracellular reactive oxygen species (ROS)

The ROS production was first observed by Immunofluorescence microscopy using MitoSOX™ Red mitochondrial superoxide indicator (lot number: M36008, Invitrogen, USA) as described before [45]. Briefly, 1-2×10^5^ cells were seeded into glass-bottom 6-well plates. After treatment, cells were incubated with MitoSOX™ reagent working solution (5 µM) for 15 min and Hoechst33258 for 15 min in the dark. The glass-bottom 6-well plates were observed using a LSM710 Laser Scanning Confocal Microscope (Zeiss, Jena, Germany).

The quantitative analysis of ROS was also measured using 2,7-dichlorofluorescin diacetate (DCFH-DA) as described previously [42]. Briefly, 10 mM DCFH-DA stock solution (in methanol) was diluted 500-fold in PBS to yield a 20 µM working solution. After MWCNT treatment, cells in each 96-well plate were washed twice with PBS and then incubated in 100 µl working solution of DCFH-DA at 37°C for 30 min. Fluorescence was determined at 485 nm excitation and 520 nm emission wavelength using an Infinite M200 microplate reader (Tecan, USA). To determine the role of ROS in the actin expression and cell migration, cells were incubated with a ROS scavenger, N-acetylcysteine (NAC) (Sigma, USA) for 2 h at 10 mM followed by MWCNT treatment. ROS level was represented as the absorbance of treated group/absorbance of control group.

### Cell scratch assay

A549 cells plated in 35-mm dishes were exposed to MWCNT at 30 µg/ml for 24 h. When cells grew to confluence, the cell monolayer was scratched to form a 100-μm “wound” using sterile pipette tips and washed gently once with PBS. A549 cells were then incubated with normal medium for another 24 h. The wound was photographed at 0 and 24 h using a TS100F microscope (Nikon, Tokyo, Japan). The cell migration distance was measured by Image J software (National Institute of Mental Health, Bethesda, USA). Data are presented as mean±standard deviation (SD) of three independent experiments.

### Cytokine assay

A commercial BD™ Cytometric Bead Array (CBA) Human Th1/Th2 Cytokine Kit II (lot number: 551809, BD Biosciences Pharmingen, San Diego, USA) was used to determine the levels (pg/ml) of Th1 and Th2 cytokines, including Interferon-gamma (IFN-γ), tumor necrosis factor (TNF), interleukin (IL)-10, IL-6, IL-4 and IL-2 in A549 supernatant samples following the manufacturer's instructions, after treatment of MWCNT at 0.3, 3, 30 µg/ml for 2, 12 and 24 h. A standard calibration curve was established for each kit. The maximum and minimum limits of detection for all six cytokines were 1.0 and 5000 pg/ml, respectively. Fluorescence was analyzed using a flow cytometer (FACS Calibur, Becton-Dickinson Biosciences, Heidelberg, Germany) and cytokine level was determined using a BD CBA Software. Statistical analysis was performed using a Kruskal-Wallis test (nonparametric analogue of one-way ANOVA) for the data that did not meet the assumptions of parametric tests. The differences were considered significant at p<0.05.

### Statistics analysis

All experiments were conducted at least three times. Statistical analysis was performed using one-way ANOVA and Student's t-test. Numerical values are represented by mean±SD. A statistical probability of p<0.05 was considered significant.

## Results

### MWCNT causes cytotoxicity following treatment for high doses and times

**Figure 1:**
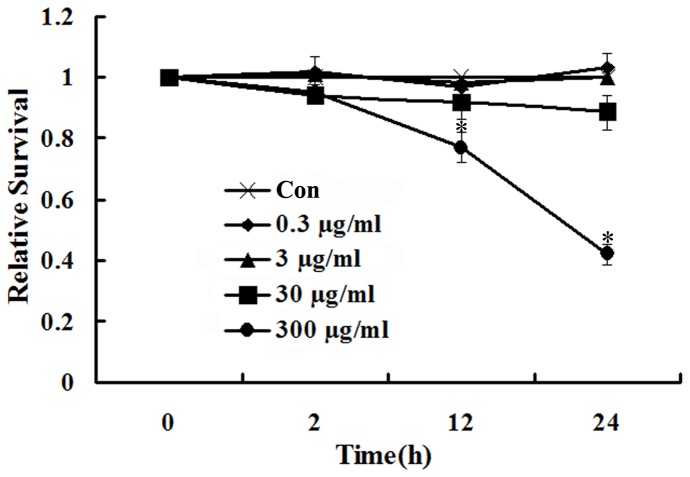
MWCNT induces cytotoxicity in A549 cells at high concentrations and longer exposure time. A549 cells were seeded into 24-well plates (1×10^4^ cells/well) and treated with MWCNT (0.3, 3, 30 and 300 µg/ml) for 2, 12 and 24 h. Cell viability was determined using the trypan blue exclusion assay.

A549 cells were treated with various concentrations (0.3, 3, 30 and 300 µg/ml) of MWCNT for the indicated times (2, 12 and 24 h), and cytotoxicity was evaluated using the trypan blue assay. As shown in Fig. 1, as compared with untreated cells, there is no significant cytotoxic effect observed for MWCNT at 0.3, 3 and 30 µg/ml throughout the 24 h period. On the other hand, at the highest concentration (300 µg/ml), the cell proliferation is significantly suppressed after treatment with MWCNT at 12 and 24 h (Fig. 1).

### MWCNT causes apoptosis and cell cycle perturbation

**Figure 2:**
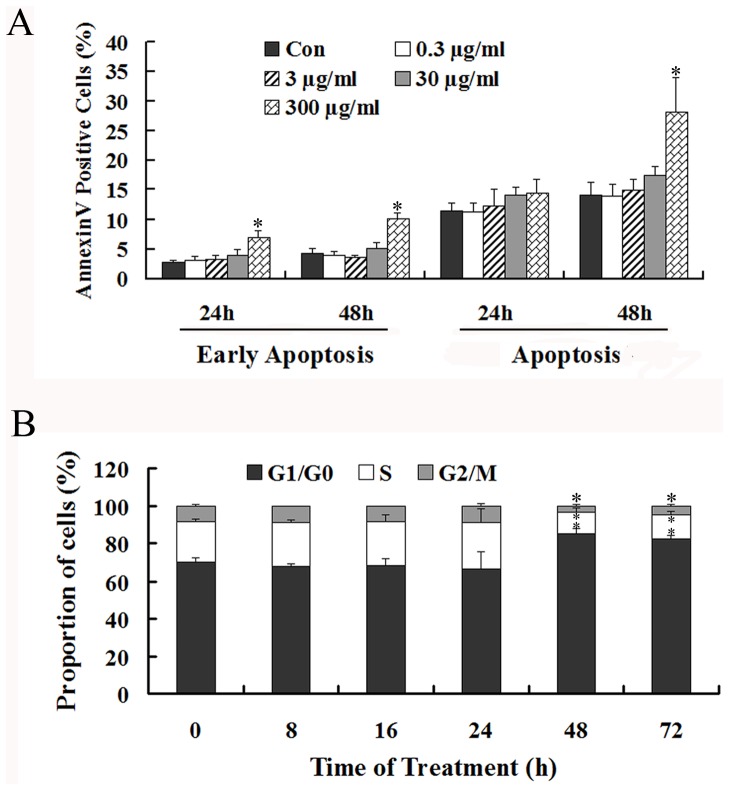
MWCNT causes apoptosis and cell cycle perturbation in A549 cells. (A) Cells were incubated with 0.3, 3, 30 and 300 µg/ml MWCNT for 24 and 48 h, and then were stained with FITC-conjugated Annexin V and PI. The percentage of early stage apoptotic cells and total apoptotic cells were calculated. (B) Cells were incubated with 30 µg/ml MWCNT for 8, 16, 24, 48 and 72 h, and then were fixed and stained with PI. Cells in G1/G0, S and G2/M phases were counted. * p<0.05, compared with control.

To determine whether apoptosis and/or cell cycle arrest had occurred, cells were examined by flow cytometry following MWCNT treatment. The results (Figure 2A) demonstrate that MWCNT fails to induce apoptosis at doses of 0.3, 3 and 30 µg/ml at 24 and 48 h. However, when treated with 300 µg/ml MWCNT for 24 h, the percentage of early apoptotic cells increases from 3% to 7% (Fig. 2A), and with prolonged exposure to 48 h, there is an approximately 2-fold increase in the percentage of both early apoptotic and apoptotic cells (Fig. 2A).

Cell cycle perturbation was also examined for cells exposed to MWCNT at 30 µg/ml for various time periods (6, 8, 24, 48 and 72 h). At shorter time periods (6, 8 and 24 h), the cell cycle distribution is almost the same for both control and treated cells (Fig. 2B). In contrast, after exposure for 48 h, the percentage of cells in G1/G0 increases significantly from 66% to 86%, while percentage of cells decreases from 9% to 4% for G2/M and 25% to 11% for S phase, respectively. Cells exposed to MWCNT for 72 h have a similar distribution throughout the cell cycle (Fig. 2B).

### MWCNT fails to demonstrate genotoxicity in A549 cells

**Figure 3:**
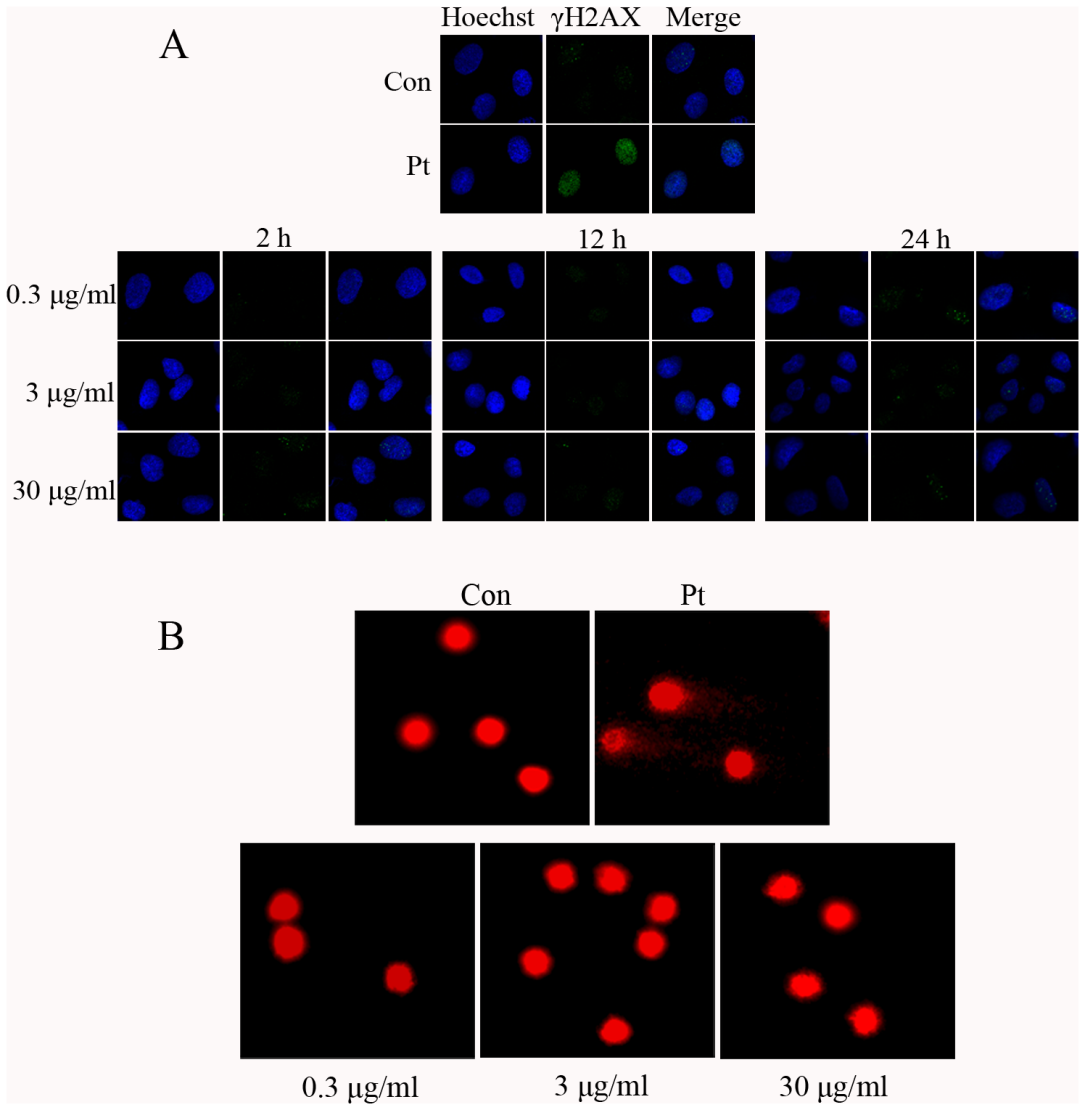
MWCNT does not cause genotoxic injury to A549 cells. (A) Following treatment with MWCNT at 0.3, 3 and 30 µg/ml for 2, 12 and 24 h, cells were fixed and stained with anti-γH2AX antibody, then subjected to immunofluorescent microscopy. Blue, Hoechst3358 stain for nuclei; Green, γH2AX. (B) Cells were treated with MWCNT for 24 h at 0.3, 3 and 30 µg/ml, and then subjected to the comet assay detection. The nucleus was stained with gel-red. 10 µM cisplatin (Pt) treated cells were used as a positive control.

To determine whether MWCNT is genotoxic to A549 cells, the effect of MWCNT on γH2AX foci formation, a sensitive indicator for DNA damage [46], was examined. Figure 3A shows representative immunofluorescent images of cells, and the results demonstrate that MWCNT treatment at 0.3, 3 and 30 µg/ml for 2, 12 or 24 h does not induce significant γH2AX foci formation.

To verify further that MWCNT indeed could not induce DNA damage, as implied by the results of our γH2AX foci formation assay, MWCNT treated cells were further subjected to the neutral comet assay. Shown in Fig. 3B are representative images from this comet assay for cells exposed to MWCNT at 0.3, 3 and 30 µg/ml for 24 h. No significant changes in tail moment or in the percentage of tail DNA, i.e., DNA damage, are observed (Table 1).

**Table 1:** Comet assay results for MWCNT treated A549 cells.

	Control	MWCNT 0.3 µg/ml	MWCNT 3 µg/ml	MWCNT 30 µg/ml	Pt
Tail DNA%	0.57±0.19	0.47±0.13	0.63±0.38	0.76±0.16	17.63±2.13*
Tail Moment	0.29±0.24	0.01±0.00	0.04±0.03	0.08±0.02	44.84±8.47*

After treatment with MWCNT at 0.3, 3 and 30 µg/ml for 24 h, the fluorescent images of A549 cells subjected to the neutral comet assay were captured and analyzed by Image-Pro software. Data are represented as mean ± SD, n = 5, * p<0.05, compared with untreated control.

### 2-DE analysis and MS identification of cellular proteins in A549 cells exposed to MWCNT

**Figure 4:**
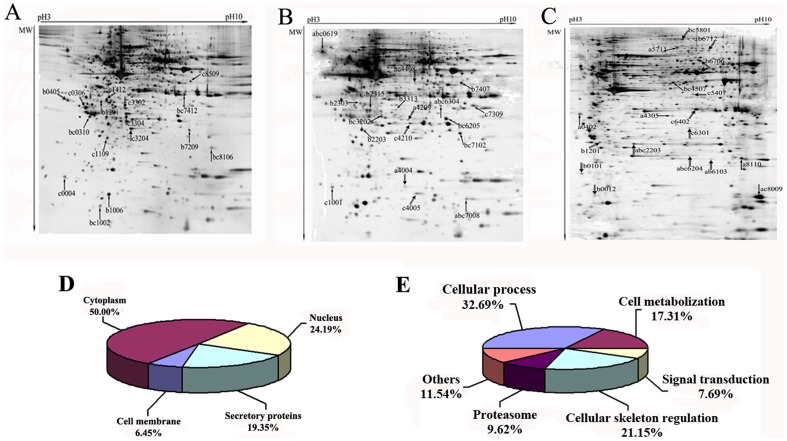
Treatment with MWCNT significantly changes protein expression. Protein extracts (200 µg) were separated on a pH 3–10, 24 cm IPG strip, followed by 12% SDS-PAGE. Proteins spots were visualized by silver staining. Shown are representative 2-DE proteomic maps of A549 cells treated with MWCNT at the indicated concentrations (a, 0.3; b, 3; c, 30 µg/ml) for various time periods (A, 2 h; B, 12 h; C, 24 h). Arrows indicate the spots identified by MS (↑up-regulated protein, down-regulated protein), and details of the corresponding spots are listed in Table 2. The location (D) and function classification (E) of identified proteins are shown in the pie charts.

Based on the results described above, A549 cells were treated with 0.3, 3 and 30 µg/ml MWCNT for 2, 12 or 24 h, and total cellular proteins were extracted and separated by 2-DE (Fig. 4A–C). After silver staining, the images of each treatment group and control were compared using PDQuest 7.4.0. In total, 106 spots show changed expression after MWCNT treatment. Of these, 62 spots were selected for LC-MS/MS identification, of which 10 spots could not be identified due to missing MS signals or low abundance. Of the 52 successfully identified spots, 29 are up-regulated and 23 are down-regulated. 50% of these proteins are located in the cytoplasm, 24.19% are located in the nucleus, and 6.45% are found on the cellular membrane, while others are mitochondrial or endoplasmic reticulum proteins (Fig. 4D). The identified proteins are involved in cellular processes such as cell cycle regulation and apoptosis, cell metabolism, and regulation of cellular skeleton, etc. (Fig. 4E and Table 2).

**Table 2:** Cellular proteins differentially regulated after MWCNT treatment.

Biological process	Spot No.	Swiss-prot ID	Protein name	Theoretical PI/MW	Function	*Fold change (p<0.05)		
						2 h	12 h	24 h
Cellular process related proteins	1201	P04632	Calpain small subunit 1	5.05/28.315.67	Cell proliferation regulation			−1.70b
	5801	Q8WUM4	Programmed cell death 6-interacting protein	6.13/96818.34	Cell apoptosis; Cell cycle regulation			−2.71b −1.88c
	6402	P04083	Annexin A1	6.57/38714.23	T cell differentiation; Anti-apoptosis			−2.33c
	8110	Q06830	Peroxiredoxin-1	8.27/22110.34	Cell proliferation; Redox regulation			+1.68a
	0619	P27797	Calreticulin	4.29/48141.7	Calcium-binding chaperone; Cell cycle arrest		+1.92a +2.37b +2.28c	
	2303 (12 h) 1412 (2 h)	P08758	Annexin A5	4.94/35936.83	Anti-apoptosis; Signal transduction	−2.24c	+1.55b	
	7309	P45880	Voltage-dependent anion-selective channel protein 2	6.32/38092.61	Form a channel to negative regulation apoptotic and protein polymerization		+1.89c	
	0306	P62258	14-3-3 protein epsilon	4.63/29173.98	G2/M transition of mitotic cell cycle; apoptotic process	−2.26c		
	1006	P09382	Galectin-1	5.34/14715.69	Regulate apoptosis, cell proliferation and cell differentiation	+1.77b		
	3204	P09211	Glutathione S-transferase P	5.43/23355.85	Cell proliferation, Glutathione metabolization	−1.50b		
	7412	P07355	Annexin A2	7.57/38604.07	Calcium binding protein, involved in heat-stress response	+2.45b +1.80c		
	4005	P31949	Protein S100-A11	6.56/11740.42	Negative regulation of DNA replication and cell proliferation		−2.29c	
	2203 (24 h) 4210 (12 h)	P04792	Heat-shock protein beta-1	5.98/22782.62	Involved in stress resistance and actin organization		−2.05c	−2.40a −2.10b −1.84c
	7102 (12 h) 7209 (2 h)	P62826	GTP-binding nuclear protein Ran	7.01/24423.06	Cell cycle; Cell division; Transport	+1.74b	+2.13b +4.40c	
Cell metabolization	7407 (12 h) 8509 (2 h)	O96008	Mitochondrial import receptor subunit TOM40 homolog	6.79/37893.05	Cellular protein metabolic process	+1.65c	+1.50b	
	4305	Q13011	Delta(3,5)-Delta(2,4)-dienoyl-CoA isomerase, mitochondrial	8.16/35816.13	Fatty acid metabolism			+1.82a
	6204	P47985	Ubiquinol-cytochrome c reductase iron-sulfur subunit, mitochondrial	8.55/29652.06	Respiratory electron transport chain			−1.59a −1.87b −2.0c
	6301	P00491	Purine nucleoside phosphorylas	6.71/32551.38	Purine nucleobase metabolic process			−1.87c
	1109	Q04760	Lactoylglutathione lyase	5.24/20719.67	Carbohydrate metabolic process; glutathione metabolic process	+1.50c		
	4209	P30084	Enoyl-CoA hydratase, mitochondrial	8.34/31387.37	Fatty acid beta-oxidation		−1.95a	
	6205	P18669	Phosphoglycerate mutase 1	6.67/28803.98	Gluconeogenesis; glycolysis		+1.60b +2.33c	
	6304	P10768	S-formylglutathione hydrolase	6.54/31462.8	Formaldehyde catabolic process		+1.84a +1.69b +1.82c	
Cell signal transduction	2315	O00299	Chloride intracellular channel protein 1	5.09/26922.76	Form chloride ion channels, involves in signal transduction		+1.56b	
	8106	P30086	Phosphatidylethanolamine-binding protein 1	7.01/21056.78	Binds ATP, opioids and phosphatidylethanolamine, as a competitive inhibitor of MEK phosphorylation	+1.50 +2.23c		
	2203 (12 h)	P52565	Rho GDP-dissociation inhibitor 1	5.03/23207.14	Rho protein signal transduction; Cellular component movement		+1.55b	
	0101	Q15185	Prostaglandin E synthase 3	4.35/18697.41	Chaperone cofactor-dependent protein refolding; signal transduction			−1.70b
Cellular skeleton	4507	P60709	Actin, cytoplasmic 1	5.29/41736.77	Structural constituent of cytoskeleton; ATP binding			+1.50b +2.40c
	5711	P26038	Moesin	6.08/67820.16	Structural constituent of cytoskeleton			+1.53a
	6706	O75083	Isoform 1 of WD repeat protein 1	6.17/66193.4	Induces disassembly of actin filaments in conjunction with ADF/cofilin family proteins			−2.49b
	8009	P23528	Cofilin-1	8.22/18502.47	Binds to F-actin, plays a role in the regulation of cell morphology and cytoskeletal organization			+1.69a +1.79c
	4408	P40121	Macrophage capping protein	5.88/38517.62	Barbed-end actin filament capping; cell projection assembly		−2.13a −2.57c	
	7008	P07737	Profilin-1	8.44/15054.14	Binds to actin and affects the structure of the cytoskeleton		+1.72a +2.27b +1.91c	
	0012	Q13182	Myosin regulatory light chain 2, nonsarcomeric	4.67/19794.13	Regulation of cell shape			−2.14b
	1001 (12 h) 0004 (2 h)	P60660	Myosin light polypeptide 6	4.56/16930.02	Actin-dependent ATPase activity	+1.72c	+2.15c	
	6712	P35908	Keratin, type II cytoskeletal 2 epidermal	8.07/65865.29	Structural constituent of cytoskeleton			−2.66c
	1301	P06753	Tropomyosin alpha-3 chain	4.75/29032.82	Implication in stabilizing cytoskeleton actin filaments		+2.21b	
Proteasome	6103	P49721	Proteasome subunit beta type 2	6.52/22836.34	DNA damage response, signal transduction by p53 class mediator resulting in cell cycle arrest; G1/S transition of mitotic cell cycle			−1.52a −2.27b
	3313	P61289	Proteasome activator complex subunit 3	5.79/30886.69	DNA damage response, signal transduction by p53 class mediator resulting in cell cycle arrest; G1/S transition of mitotic cell cycle		+2.22b	
	4004	P61088	Ubiquitin-conjugating enzyme E2 N	6.13/17137.86	DNA damage; DNA repair; Ubl conjugation pathway		−2.84a	
	0310	P28066	Proteasome subunit alpha type-5	4.74/26411.05	DNA damage response, signal transduction by p53 class mediator resulting in cell cycle arrest; G1/S transition of mitotic cell cycle	+1.51b +1.68c		
	3302	Q86SZ7	Proteasome activator subunit 2	5.54/27401.69	Proteasome activator complex	−1.85c		
Others	5407	Q15366	Poly(rC)-binding protein 2	6.33/38221.66	Innate immune response; mRNA splicing via spliceosome			−2.03c
	3202 (12 h) 3304 (2 h)	P40261	Nicotinamide N-methyltransferase	5.56/29574.1	Catalyzes the N-methylation of nicotinamide and other pyridines to form pyridinium ions	−2.36c	+1.87b +2.35c	
	0405	Q07021	Complement component 1 Q subcomponent-binding protein, mitochondrial	4.74/31362.33	Involved in inflammation and infection processes, ribosome biogenesis, regulation of apoptosis, transcriptional regulation and pre-mRNA splicing	−2.12b		
	1002	P61970	Nuclear transport factor 2	5.1/14478.47	Facilitates protein transport into the nucleus	+1.97b +1.92c		
	0402	P43307	Translocon-associated protein subunit alpha	4.39/32235.45	Involves in the recycling of the translocation apparatus			+1.51a

+, up-regulated proteins; -, down-regulated proteins; a, 0.3 µg/ml; b, 3 µg/ml; c, 30 µg/ml.

### Validation of identified proteins

**Figure 5:**
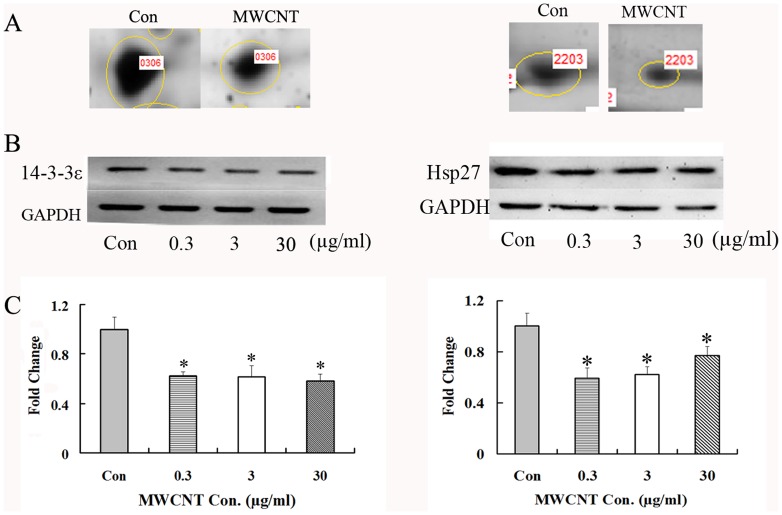
MWCNT treatment decreases 14-3-3ε and HSP27 expression. (A) Enlarged 2-DE images of spot 0306 (left panel) and spot 2203 (right panel), which were down-regulated in MWCNT-treated A549 cells; (B) Western blot results for 14-3-3ε (left panel)/HSP27 (right panel) expression in A549 cells treated with 0.3, 3 and 30 µg/ml for 2 h/24 h; (C) Densitometry analysis of (B). GAPDH was used as a loading control. * p<0.05.

**Figure 6:**
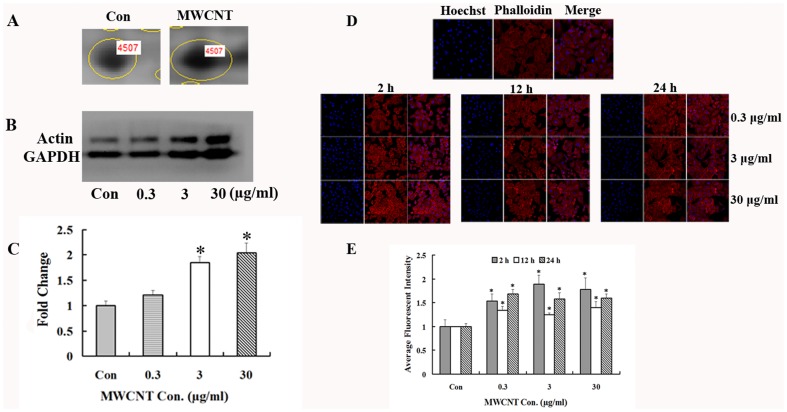
MWCNT treatment increases actin expression. (A) Enlarged 2-DE images of spot 4507, which was up-regulated in MWCNT-treated A549 cells. (B) Western blot results for actin expression in A549 cells treated with 0.3, 3 and 30 µg/ml for 24 h. (C) Densitometry analysis of (B). GAPDH was used as a loading control, * p<0.05. (D) Immunofluorescence microscopic analysis of actin expression after MWCNT treatment. After treatment with MWCNT at 0.3, 3 and 30 µg/ml for 2, 12 and 24 h, cells were fixed and stained with phalloidin, then subjected to immunofluorescence microscopy. Shown are representative images from one of three independent experiments. Blue, Hoechst3358 stain for nuclei. Red, Actin. 10 µM Pt was used as a positive control. (E) Semi-quantification data of the average fluorescent intensity. * p<0.05, compared to control.

To verify the proteomic results, Western blot was used to confirm the expression changes of three identified proteins, namely, 14-3-3ε, HSP27 and actin. The results support the proteomic results, showing that MWCNT treatment induces a significant decrease in 14-3-3ε (Fig. 5, left panel) and HSP27 (Fig. 5, right panel) at the indicated concentrations. On the other hand, actin is up-regulated after 24 h of treatment (Fig. 6A–C). Each of these results is consistent with the proteomic analysis.

To further confirm these results, confocal microscopy imaging analysis was applied to investigate the effects of MWCNT on actin expression in A549 cells. Cells were exposed to 0.3, 3 and 30 µg/ml MWCNT for the indicated times and the expression level of actin filaments was determined by phalloidin staining. As shown in Fig. 6D–E, the fluorescent intensity of actin is significantly increased after exposure to MWCNT, consistent with our Western blot results.

### ROS modulates the changes of protein expression induced by MWCNT

**Figure 7:**
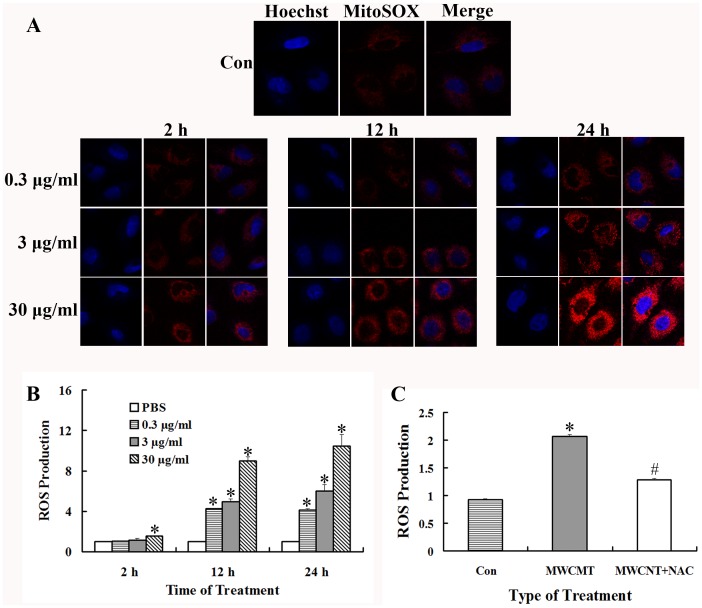
MWCNT induces the generation of cellular reactive oxygen species (ROS). (A) Cells were exposed to 0.3, 3 and 30 µg/ml MWCNT for 2, 12 and 24 h, and ROS generation was measured using MitoSOX™ Red mitochondrial superoxide indicator by Immunofluorescence microscopy. (B) Cells were exposed to the indicated concentrations of MWCNT for various times, and ROS generation was measured using 2′,7′-dichlorofluorescin diacetate (DCFH-DA) using a spectrofluorometer. ROS generation was expressed as fold increase of fluorescence compared to the control; * p<0.05, compared to PBS treated control. (C) Cells were pretreated with NAC (10 mM) for 2 h, and then exposed to 30 µg/ml of MWNCT for 24 h. * p<0.05, compared with the control. # p<0.05, compared to MWCNT treatment without NAC pre-incubation.

It has been reported that many of the bioeffects of MWCNT can be mediated through the changes of cellular oxidative status [14], [19], [20]. To determine whether MWCNT could affect ROS production in A549 cells, MitoSOX, a novel fluoroprobe, was introduced for selective detection of superoxide in the mitochondria of live cells. Shown in Fig. 7A are the representative images of cells after MWCNT treatment, which indicated that mitochondrial ROS was increased in a time- and dose-dependent manner. The intracellular ROS levels were also quantitatively measured by DCFH-DA staining. The results show that the ROS levels are significantly increased after exposure to MWCNT at 12 and 24 h (Fig. 7B), which is consistent with the confocal microscopy results.

**Figure 8:**
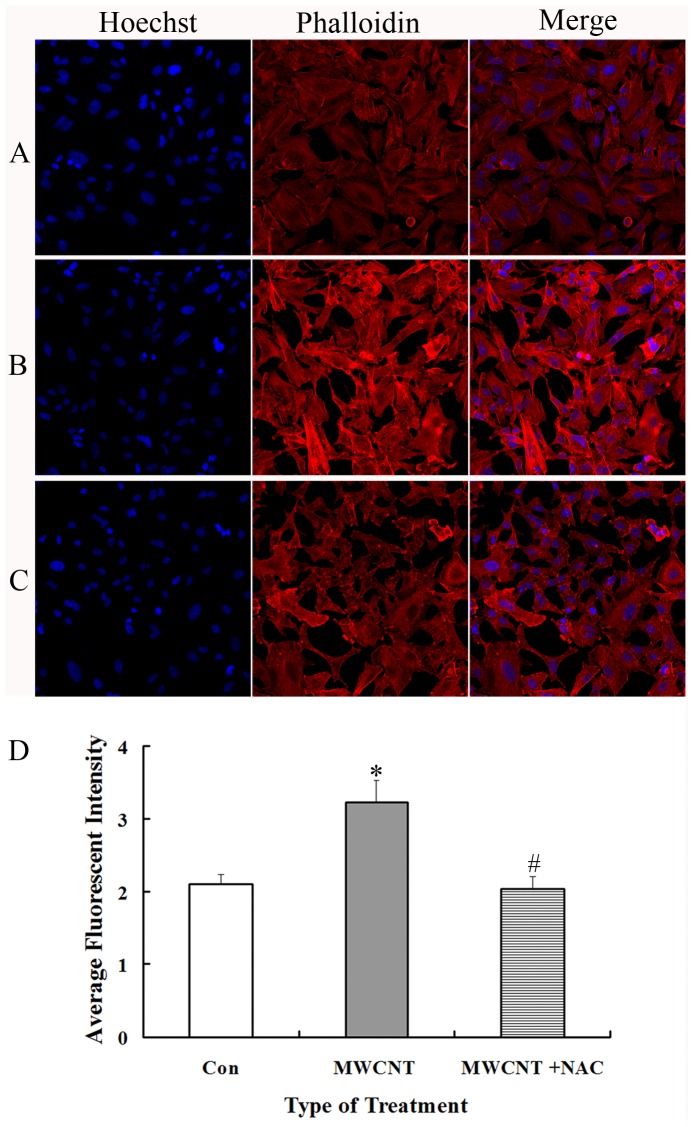
Inhibition of ROS generation attenuates actin expression in MWCNT-treated A549 cells. After MWCNT treatment, cells were fixed and stained with phalloidin, and then subjected to immunofluorescent microscopy. (A) Control; (B) Cells treated with 30 µg/ml of MWNCT for 24 h; (C) Cells treated with NAC (10 mM, 2 h) followed by 30 µg/ml of MWNCT for 24 h; (D) Quantitative analysis of average fluorescent intensity of the above images. * p<0.05, compared with the control; # p<0.05, compared with MWCNT treatment cells.

Next, to determine whether ROS is involved in MWCNT-induced increases in actin expression, A549 cells were pretreated with an antioxidant NAC (10 mM, 2 h) before exposure to MWCNT. As anticipated, pre-treatment with NAC dramatically decreases the level of ROS (Fig. 7C). Consistent with the hypothesis that MWCNT-mediated increases in actin expression are mediated through ROS, we find that actin expression is significantly decreased after NAC-pretreatment as compared to MWCNT exposure only (Fig. 8).

### MWNCT promotes cell migration

**Figure 9:**
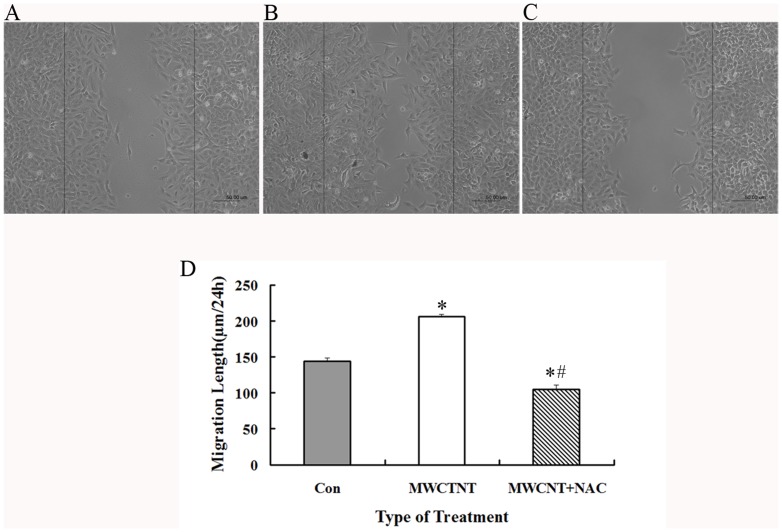
MWNCT leads to increased cell migration. A549 cells were grown to confluence, scratched, and allowed to recover for 24(A) Control; (B) 30 µg/ml MWCNT exposure; (C) Cells treated with NAC (10 mM, 2 h) followed by 30 µg/ml of MWNCT for 24 h. Shown were representative images from three independent experiments. Scale bar = 50 µm. (D) The distances of migrating cells were determined and data were presented as mean ± SD. * p<0.05 versus control, # p<0.05 versus MWCNT treated cells.

As described above, MWCNT exposure induces ROS generation and actin expression, and both are known to be directly related to the ability of cells to migrate [21], [47]. Therefore, the effects of MWCNT on cell migration were measured by cell scratch analysis. It is found that cell migration ability is indeed increased by MWCNT exposure (Fig. 9A–B). Moreover, the pre-treatment by NAC, which inhibits the production of ROS, also leads to decreased cell migration (Fig. 9C), indicating the involvement of ROS during this process.

### The effects of MWCNT on Th1/Th2 cytokine production

**Figure 10:**
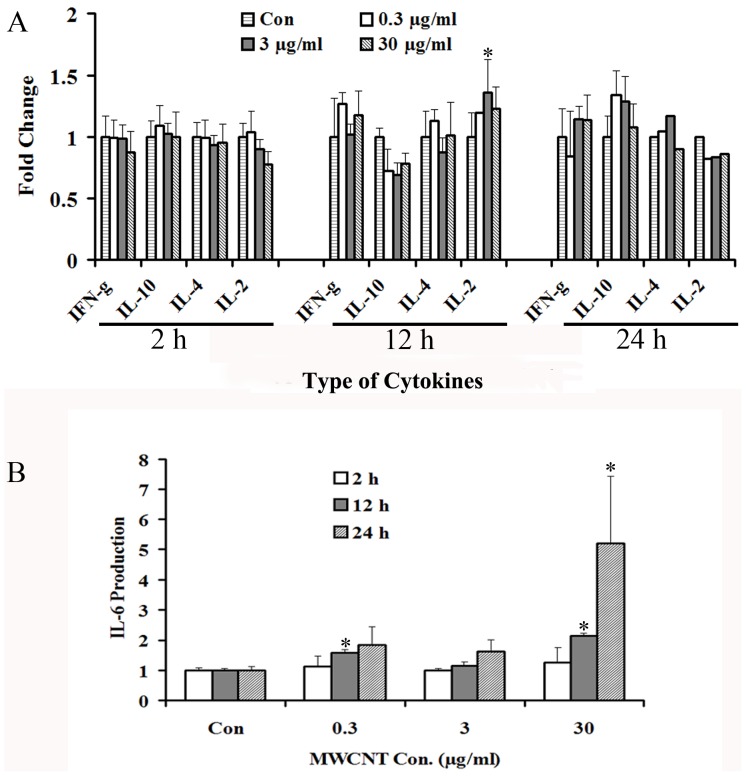
Effects of MWCNT exposure on the expression of Th1/Th2 cytokines. Following treatment with MWCNT at 0.3, 3 and 30 µg/ml for 2, 12 and 24 h, the supernatants of A549 cells were collected and detected using a Human Th1/Th2 Cytokine Kit II by flow cytometry. Shown are expression levels of IFN-γ, IL-10, IL-4, IL-2 (A) and IL-6 (B).

A549 cells were treated with various concentrations (0.3, 3 and 30 µg/ml) of MWCNT for the indicated times (2, 12 and 24 h), and the secretion of Th1/Th2 cytokines were meausred in the supernatant. TNF is not detected, probably due to the detection limit of the kit. For IFN-γ, IL-2, IL-10, and IL-4 secretion, as shown in Fig. 10A, no significant changes are found after MWCNT treatment. In contrast, IL-6 secretion is affected by MWCNT treatment, which is increased in a time- and dose-dependent manner (Fig. 10B).

## Discussion

The increasing use of MWCNT in consumer products and medical applications underlines the importance of understanding its potential toxic effects to human and the environment. However, the toxicity and related molecular mechanisms of MWCNT have not been fully elucidated, with many contradictive results reported over the years. Thus, in the present study, we examined the effects of MWCNT on A549 cells. In particular, we employed a proteomic approach in an effort to provide a systematic view of the cellular response to MWCNT exposure.

We first find that MWCNT causes cytotoxic injuries to A549 cells only at relatively high concentrations and longer exposure time, which appears to be relatively more resistant to MWCNT exposure as compared to other cell types. In our study, after exposure to 30 µg/ml MWCNT for 24 h, no significant changes in cell cycle, cell apoptosis, or DNA damage are detected. This is consistent with previous reports showing that no significant suppression of A549 cell proliferation could be detected after exposure to 5-100 µg/ml MWCNT for 24 h [21], [24]. On the other hand, in a previous study we had shown that exposure of the same MWCNT to human umbilical vein endothelial cell (HUVEC) significantly increases the percentage of apoptotic cells and γH2AX-positive cells in a dose-dependent manner (0.5–20 µg/ml) [12]. Human skin fibroblast cells (HSF) are even more sensitive, as exposure to the exact same MWCNT at a dose of only 0.06 µg/ml for 48 h could result in 50% reduction in proliferation, a 2-fold increase in apoptosis/necrosis and G2/M block [14]. Still, Ursini et al. reports that significant cell death and DNA damage occur after 20 µg/ml MWCNT treatment of A549 cells for 24 h [48]. These contradictory observations could be the results of different MWCNT materials, cell lines, detection methods and protocols in individual laboratories. Thus, a more comprehensive and standardized evaluation of the cytotoxic effects of MWCNT is warranted in order to obtain a clearer answer.

High-throughput and systems-based technologies, such as proteomics, can reveal complex interactions in biological systems and thereby provide new leads for mechanistic study [49]. Therefore, we examined the cellular response to MWCNT at dosages of 0.3, 3 and 30 µg/ml for 2, 12 and 24 h, for which no significant cytotoxicity, apoptosis, cell cycle perturbation, and DNA damage are induced. Eventually, 106 proteins with altered expression are detected, of which 52 are successfully identified. These 52 proteins are involved in various biological processes, including apoptosis, cell cycle arrest, cell metabolization and cellular skeleton regulation. Some of the identified proteins, such as HSP27, Annexin A2, 14-3-3ε and proteasome components, match those reported by Haniu, et al, who analyzed the proteome response by exposing U937 cells to MWCNTs at non-cytotoxic dosages [50]. Similar functional perturbation of cellular processes like apoptosis, stress, metabolism, and cytoskeleton are also reported following MWCNT treatment of RAW264.7 macrophage cells and human epidermal keratinocytes (HEKs) [51], [52], and in human hepatoma HepG2 cells treated with single-walled carbon nanotubes (SWCNT) [53].

Among the identified proteins, 21.15% are cellular skeleton proteins such as actin, confilin and profilin. Actin is the major component of microfilaments, and its structural modulation is the basis of molecular adhesion, cellular communication, cell permeability changes and cell movement [54]–[56]. Alterations in actin can be triggered by many external events, including HIV infection [57]. In one previous study, exposure to 1.5–4.5 µg/ml of MWCNT causes actin filament disruption and reduced tubule formation in human aortic endothelial cells [58]. In another study, MWCNT induces actin filament remodeling to form peripheral motile structures, lamellipodia and filopodia, and central actin filament bundles in human microvascular endothelial cells (HMVEC), and the cells are pulled apart to form small gaps in the HMVEC monolayer [59]. Mechanistically, reports have revealed that the elevation of ROS levels might be responsible for changes in the actin structure [59]–[62]. In our study, MWCNT causes significant increases in actin expression under all treatments, along with elevated ROS levels. Following pre-treatment with NAC for 2 h, ROS levels are decreased, followed by a significant decrease of actin expression in A549 cells. These results indicate that ROS is likely involved in MWCNT-induced actin alterations, consistent with previous reports.

Cell migration is an important process under several physiological conditions like development and wound healing, and also under the pathological conditions such as cancer cell invasion and metastasis [63]. Migration involves a wide array of cellular changes including alterations in cellular structure by regulation of cytoskeleton dynamics and expression of adhesion molecules [64]. Therefore, changes in actin structure have the ability to directly affect cell migration [47], [65], [66]. ROS are also known to actively participate in each of the above events [67]–[69]. Since MWCNT significantly induces the generation of ROS and actin expression, it is of interest to evaluate the effects of MWCNT on cell migration. The cell scratch assay results clearly show that MWCNT exposure can increase cell migration, while pre-treatment of NAC abolishes this effect, which are consistent with other studies regarding the effects of MWCNT on cell migration [59], [70].

It has been reported that MWCNT could trigger inflammatory response in mice or cell lines [31], [33], and the increased ROS production is at least partially responsible for it [71], [72], although contradictive results also exist [23], [41]. In our study, several proteins involved in inflammation and infection processes, such as Annexin A1, Complement component 1 Q subcomponent-binding protein (C1qBP), Poly(rC)-binding protein 2 (hnRNP E2) are indentified (Table 2). Additionally, we also find an elevated ROS production. Therefore, it is of interest to know whether MWCNT could influence the inflammatory response in A549 cells. Among the Th1/Th2 cytokines examined, only IL-6 secretion is affected by MWCNT treatment. Thus, the IL-6-mediated inflammatory response might be a major target of MWCNT.

Several of the other identified proteins are also of interest for further study. 14-3-3ε is the most conserved member of the 14-3-3 family, and is involved in a wide range of physiological processes. For example, homocysteine induces apoptosis of rat hippocampal neurons by inhibiting 14-3-3ε expression [73], and down-regulation of 14-3-3ε causes partial meiotic resumption of the mouse oocyte [74]. Moreover, a significant reduction of 14-3-3ε protein expression is detected in gastric cancer [75]. Given the prevalence of reduced 14-3-3ε expression in such processes, the importance of reduced 14-3-3ε expression after MWCNT exposure merits further investigation. Another interesting protein is Hsp27, a member of the small heat shock protein (HSP) family, which protects against apoptotic cell death induced by a variety of stimuli including elevated temperature, heavy metals, oxidative stress and cytotoxic agents [76], [77]. Our initial prediction is that under stress conditions, such as MWCNT exposure, HSP expression would have been induced for its protective function, however, we find instead that HSP27 expression is decreased. The physiological significance of such changes requires further clarification.

## Conclusions

In the present study, we demonstrate that MWCNT induces cytotoxicity in A549 cells only at relatively high concentrations and longer exposure time. Within a relatively low dosage range (30 µg/ml) and short time period (24 h), MWCNT treatment of A549 cells does not induce significant cytotoxicity, cell cycle arrest, cell apoptosis, or DNA damage. However, under the same treatment condition, MWCNT causes significant changes in protein expression. The differentially expressed proteins could provide new leads for deciphering the cellular response to MWCNT. As one example, MWCNT treatment causes increased actin expression, likely mediated by ROS and leading to increased migration capacity.
